# Targeted next-generation sequencing and long-read HiFi sequencing provide novel insights into clinically significant KLF1 variants

**DOI:** 10.1186/s12864-024-10148-x

**Published:** 2024-03-01

**Authors:** Luyi Ye, Chen Wang, Aijing Li, Minghao Li, Yan Pi, Jingmin Yang, Ziyan Zhu, Daru Lu

**Affiliations:** 1 State Key Laboratory of Genetic Engineering and MOE Engineering Research Center of Gene Technology, School of Life Sciences, Fudan University, 200433 Shanghai, China; 2 Shanghai Institute of Blood Transfusion, Shanghai Blood Center, 20051 Shanghai, China; 3 NHC Key Laboratory of Birth Defects and Reproductive Health (Chongqing Key Laboratory of Birth Defects and Reproductive Health, Chongqing Population and Family Planning Science and Technology Research Institute), 400020 Chongqing, China; 4 Shanghai WeHealth Biomedical Technology Co., Ltd, 201318 Shanghai, China

**Keywords:** KLF1, In(Lu) phenotype, LU blood group, NGS, HiFi sequencing

## Abstract

**Background:**

Krüppel-like factor 1 (KLF1), a crucial erythroid transcription factor, plays a significant role in various erythroid changes and haemolytic diseases. The rare erythrocyte Lutheran inhibitor (In(Lu)) blood group phenotype serves as an effective model for identifying KLF1 hypomorphic and loss-of-function variants. In this study, we aimed to analyse the genetic background of the In(Lu) phenotype in a population-based sample group by high-throughput technologies to find potentially clinically significant KLF1 variants.

**Results:**

We included 62 samples with In(Lu) phenotype, screened from over 300,000 Chinese blood donors. Among them, 36 samples were sequenced using targeted Next Generation Sequencing (NGS), whereas 19 samples were sequenced using High Fidelity (HiFi) technology. In addition, seven samples were simply sequenced using Sanger sequencing. A total of 29 hypomorphic or loss-of-function variants of KLF1 were identified, 21 of which were newly discovered. All new variants discovered by targeted NGS or HiFi sequencing were validated through Sanger sequencing, and the obtained results were found to be consistent. The *KLF1* haplotypes of all new variants were further confirmed using clone sequencing or HiFi sequencing. The lack of functional KLF1 variants detected in the four samples indicates the presence of additional regulatory mechanisms. In addition, some samples exhibited *BCAM* polymorphisms, which encodes antigens of the Lutheran (LU) blood group system. However, no *BCAM* mutations which leads to the absence of LU proteins were detected.

**Conclusions:**

High-throughput sequencing methods, particularly HiFi sequencing, were introduced for the first time into genetic analysis of the In(Lu) phenotype. Targeted NGS and HiFi sequencing demonstrated the accuracy of the results, providing additional advantages such as simultaneous analysis of other blood group genes and clarification of haplotypes. Using the In(Lu) phenotype, a powerful model for identifying hypomorphic or loss-of-function KLF1 variants, numerous novel variants have been detected, which have contributed to the comprehensive understanding of KLF1. These clinically significant KLF1 mutations can serve as a valuable reference for the diagnosis of related blood cell diseases.

**Supplementary Information:**

The online version contains supplementary material available at 10.1186/s12864-024-10148-x.

## Background

Transcription factors are crucial in the formation of cell-specific regulatory networks. Mutations in transcription factors or transcription factor binding sites have been identified to be associated with various human diseases [1]. In addition, transcription factors are significant in hematopoietic lineage differentiation and hematopoietic stem cell homeostasis. Therefore, they are potential therapeutic targets for haematological diseases [2, 3].

KLF1, also referred to as Erythroid Krüppel-like factor (EKLF), is a crucial transcription factor involved in erythroid development. KLF1 is exclusively expressed in hematopoietic organs, which plays a vital role in erythroid lineage commitment, haemoglobin conversion, erythropoietic terminal differentiation and the expression of erythrocyte antigens [4–6]. Given its critical role in various physiological events, naturally occurring KLF1 mutations had been believed to be extremely rare initially until the first mutation of human *KLF1* gene was identified in an individual with the In(Lu) phenotype in 2008 [7]. KLF1 mutations have been found to cause different forms of anaemia and a range of red blood cell disorders, including various unrelated haemoglobin abnormalities [8].

The In(Lu) rare blood group, presenting a down-regulated expression level of multiple blood group antigens, is primarily due to heterozygous clinically significant KLF1 mutation [6]. The major antigens affected in the In(Lu) phenotype are those of the Lutheran (LU) blood group system, which expression is significantly suppressed. In addition, the expression of antigens in other blood group systems such as IN, P1PK, LW and KN is inhibited to varying degrees, but individual differences are observed in the degree of down-regulation and whether or not such expression levels of antigens were down-regulated [6]. Thus, individuals with the rare In(Lu) phenotype can be screened out by detecting LU antigens, and then such individuals can be detected to find functional KLF1 mutations.

The LU blood group system is encoded by the *BCAM* gene, comprising 27 antigens that are carried by the basal cell adhesion molecule (BCAM), which is also known as CD239 [9]. Lu^a^ and Lu^b^ are the primary antithetical antigens in the LU blood group system, which are determined by the polymorphism c.230G > A (rs28399653) in exon 3 of the *BCAM* gene. Three rare null or mod phenotypes in the LU system have been reported, namely Lu_null_, In(Lu), and XS2, with similar serological features [10]. The Lu_null_ or Lu(a − b−) phenotype, which is characterized by the absence of all Lutheran system antigens, is caused by the inactivation of the *BCAM* gene. In the dominantly inherited In(Lu) phenotype mainly caused by *KLF1* mutations, the expression of BCAM reduces to extremely low levels, making it almost impossible to detect LU antigens using serological agglutination tests. The X-linked XS2 phenotype is caused by hemizygous variants in the *GATA1* gene, which is also not usually detectable by serological agglutination methods [10]. Therefore, comprehensive genetic testing can provide a more complete picture to determine the In(Lu) phenotype.

In this study, we analysed the genetic background of serological Lu(a − b−) blood samples collected over the past 10 years, taking the appropriate sequencing strategy in accordance with DNA quality measurements. The results enriched our understanding of potentially pathogenic KLF1 mutations and highlighted the advantages of utilising high-throughput sequencing technologies in solving complex genotype–phenotype problems.

## Results

### Serotypes

From over 300,000 blood donors, a total of 62 blood samples that tested negative for anti-Lu^a^ and anti-Lu^b^ were screened out and collected in this study, with apparently Lu(a − b−) phenotype. DNA extracts from these samples were stored and scheduled for subsequent genetic testing to confirm the In(Lu) phenotype and analyse the genetic background.

### Identification of KLF1 mutations and other polymorphisms

**Fig. 1:**
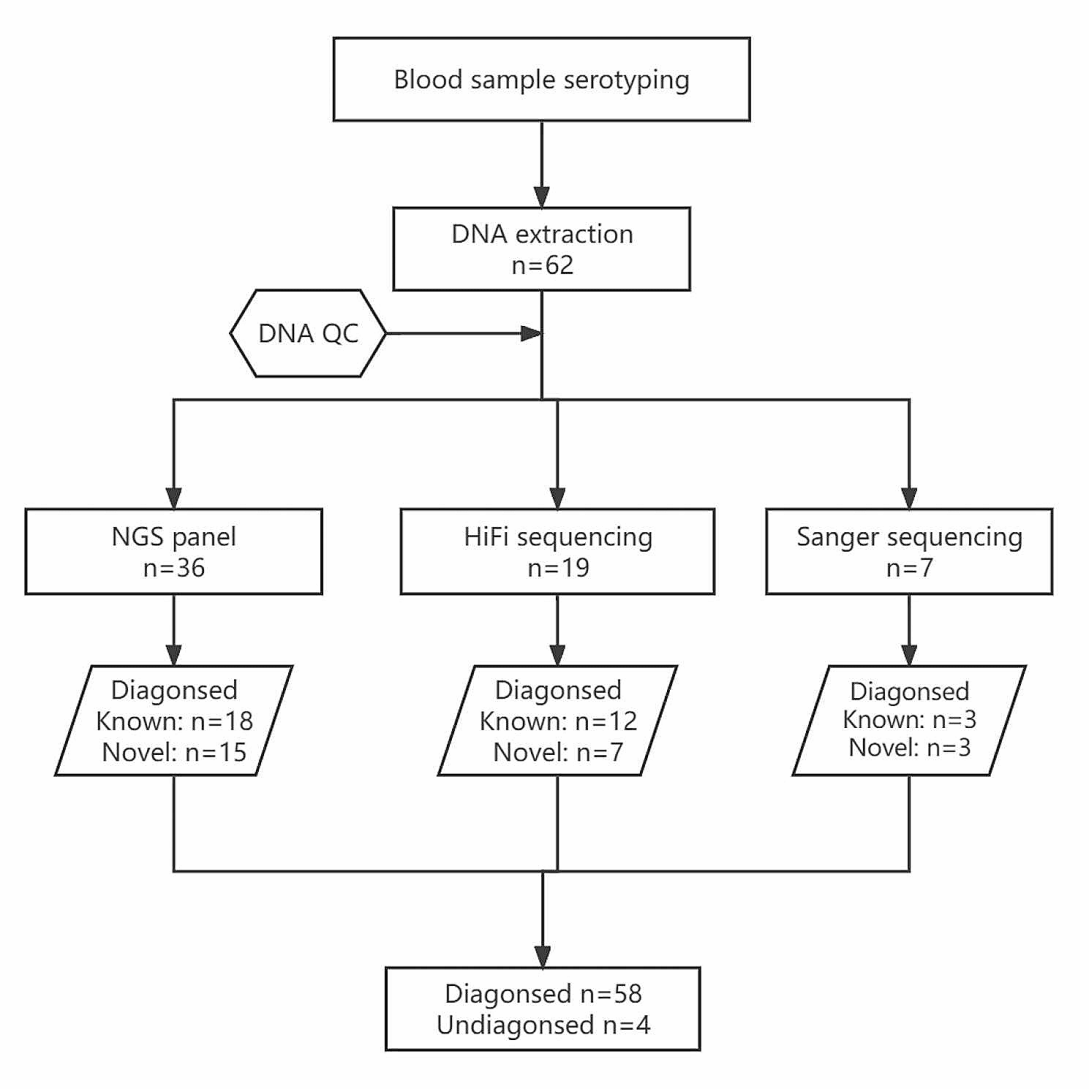
Diagram of simplified sample collection and sequencing process

**Fig. 2:**
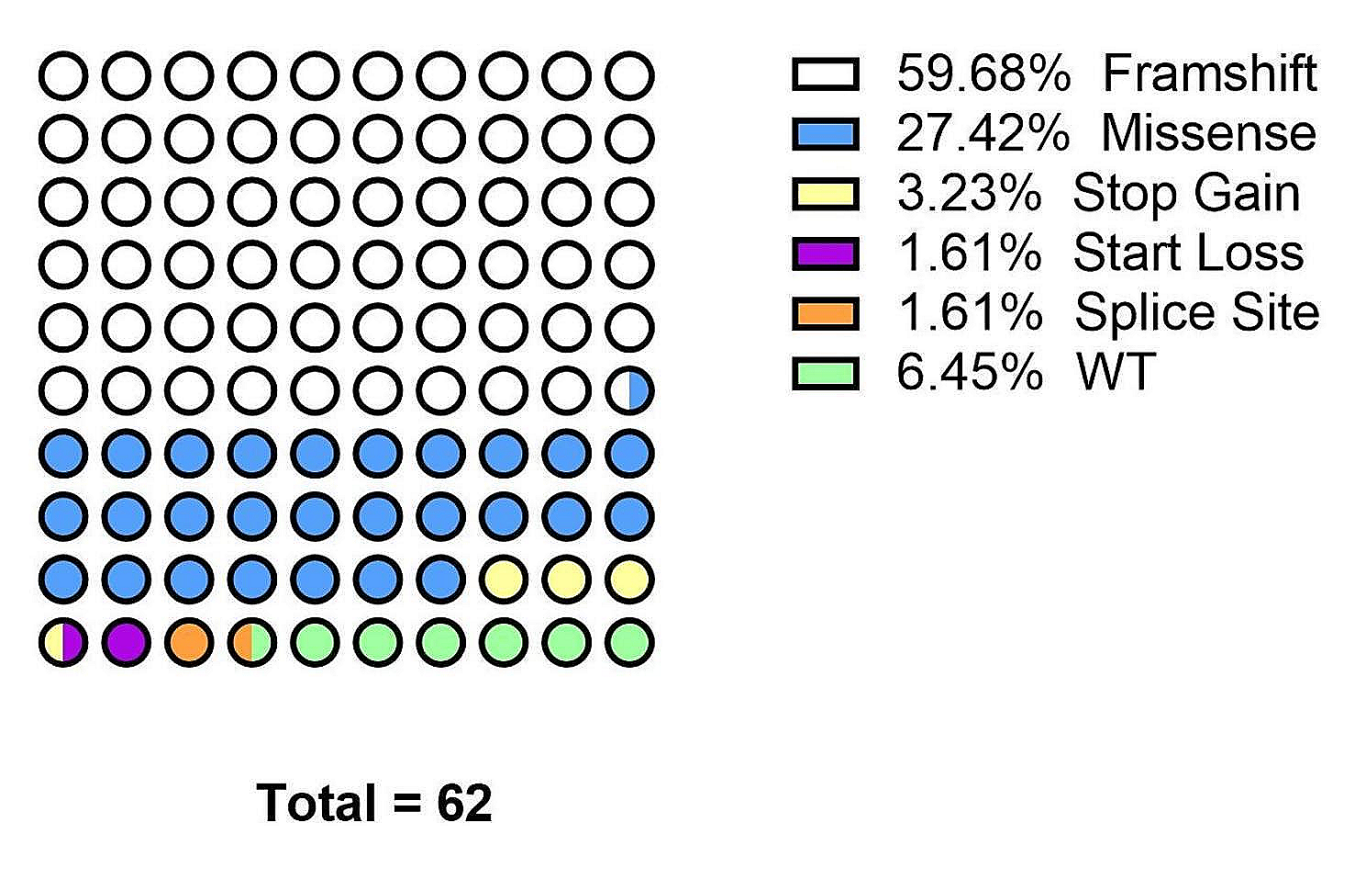
The types and percentages of *KLF1* mutations in 62 samples

**Fig. 3:**
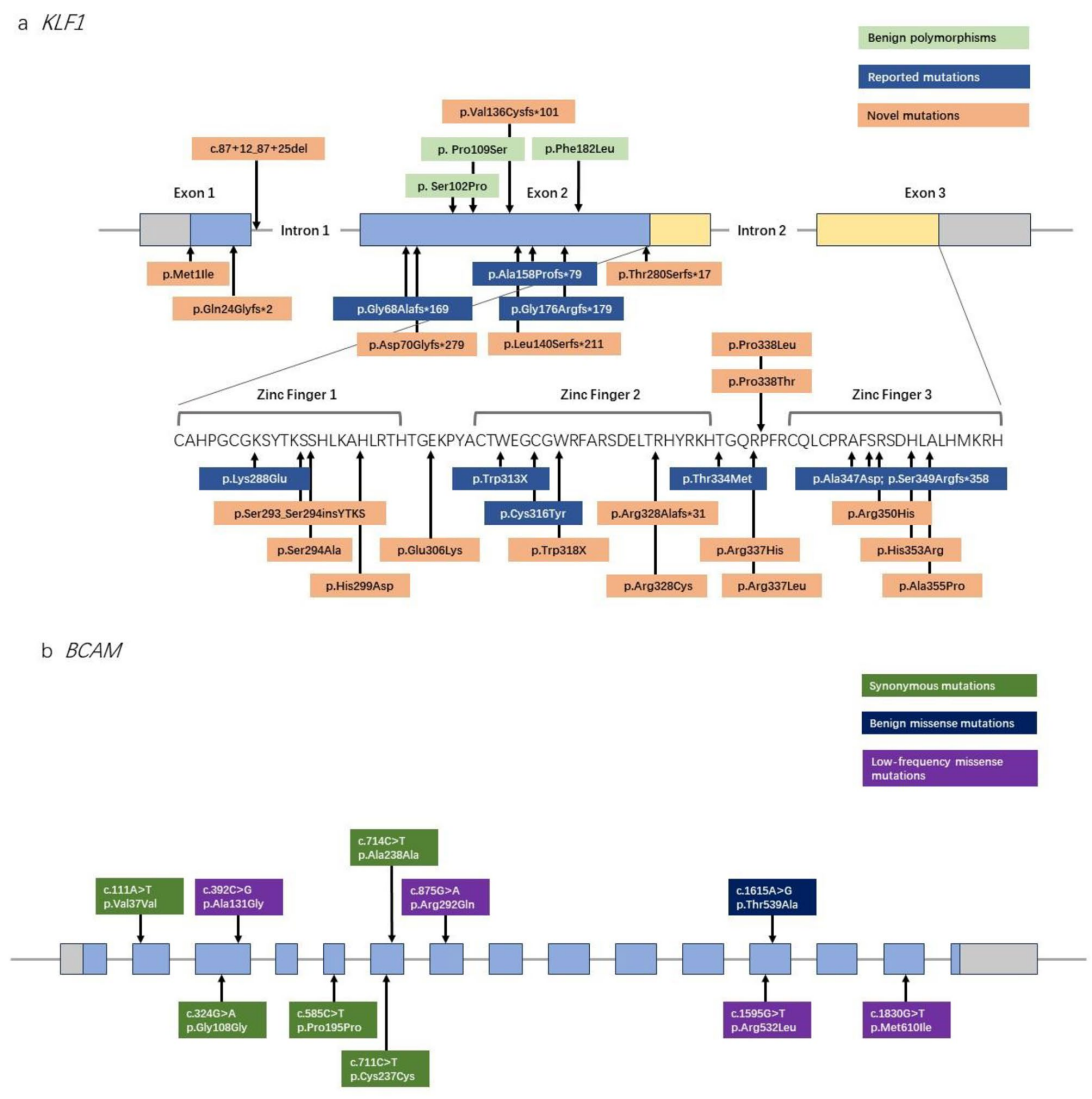
The polymorphisms and mutations of KLF1 and BCAM identified in high-throughput sequencing samples. a, schematic diagram of the *KLF1* gene. Boxes, exons; gray, non-coding regions; blue, coding regions; yellow, zinc fingers. b, schematic diagram of the *BCAM* gene. Boxes, exons; gray, non-coding regions; blue, coding regions

Out of the total 62 samples, high-throughput sequencing (NGS or HiFi) was performed on 55 samples according to DNA quality. The schematic diagram of sequencing detection is shown in Fig. 1. Detailed information of all identified KLF1 mutations is illustrated in Table 1. The direct and clone sequencing peak maps of 21 novel *KLF1* variants are shown in Additional file 1. No pathological mutations of the *KLF1* gene were detected in four samples. The *KLF1* mutation types and percentages of 62 samples are displayed in Fig. 2. Figure 3 presents the information regarding the polymorphisms and mutations of KLF1 and BCAM identified in all the samples subjected to high-throughput sequencing. Moreover, no loss-of-function mutations in other blood group genes were observed in any of the NSG samples, and no structural variations were detected in the *BCAM* and *KLF1* genes in any of the HiFi samples.

**Table 1:** *KLF1* mutations identified in In(Lu) blood samples

Type	Nucleotide change	Exon/Intron	Predicted amino acid change	Mutation Type	Allele Name	Number of samples	Identified by	Ref/GenBank No.
reported	c.519_525dupCGGCGCC	Exon 2	p.Gly176Argfs*179	Frameshift	KLF1*BGM34	24	NGS 10; HiFi 11; Sanger 3	Ref 9
	c.199delA	Exon 2	p.Gly68Alafs*169	Frameshift	KLF1*BGM64	1	NGS	Ref 9
	c.472delG	Exon 2	p.Ala158Profs*79	Frameshift	KLF1*BGM52	1	NGS	Ref 9
	c.862 A > G	Exon 2	p.Lys288Glu	Missense	KLF1*BGM37	1	HiFi	Ref 9
	c.939G > A	Exon 3	p.Trp313X	Stop Gain	KLF1*BGM55	1	NGS	Ref 9
	c.947G > A	Exon 3	p.Cys316Tyr	Missense	KLF1*BGM29	1	NGS	Ref 9
	c.1001 C > T	Exon 3	p.Thr334Met	Missense	KLF1*BGM48	2	NGS	Ref 9
	c.1040 C > A; c.1045delT	Exon 3	p.Ala347Asp; p.Ser349Argfs*358	Missense; Frameshift	KLF1*BGM19	2	NGS	Ref 9
novel	c.3G > A	Exon 1	p.Met1Ile	Start Loss		1	HiFi	OQ054249
	c.70_71delCA	Exon 1	p.Gln24Glyfs*2	Frameshift		1	NGS	OQ716571
	c.87 + 12_87 + 25delAAGGTGGGGTCTAG	Intron 1	NA	Splice Site		1	NGS	OR000215
	c.207_217delGGACGCCACCT	Exon 2	p.Asp70Glyfs*279	Frameshift		1	HiFi	OR000213
	c.406delG	Exon 2	p.Val136Cysfs*101	Frameshift		2	NGS 1; Sanger 1	OQ716569
	c.417_421delCCTGC	Exon 2	p.Leu140Serfs*211	Frameshift		1	HiFi	OQ716565
	c.839delC	Exon 2	p.Thr280Serfs*17	Frameshift		1	NGS	OR000214
	c.880_881insACACCAAGAGCT	Exon 2	p.Ser293_Ser294insYTKS	Frameshift		1	NGS	OQ716572
	c.880T > G	Exon 2	p.Ser294Ala	Missense		1	NGS	OQ054248
	c.895 C > G	Exon 2	p.His299Asp	Missense		1	Sanger	OQ716563
	c.916G > A	Exon 3	p.Glu306Lys	Missense		2	NGS 1; Sanger 1	OQ054251
	c.953G > A	Exon 3	p.Trp318X	Stop Gain		1	HiFi	OR000212
	c.982delC	Exon 3	p.Arg328Alafs*31	Frameshift		2	NGS	OQ716568
	c.982 C > T	Exon 3	p.Arg328Cys	Missense		1	NGS	OQ054250
	c.1010G > A	Exon 3	p.Arg337His	Missense		1	HiFi	OQ054253
	c.1010G > T; 1011 C > T	Exon 3	p.Arg337Leu	Missense		1	NGS	OQ716566
	c.1012 C > A	Exon 3	p.Pro338Thr	Missense		2	NGS	OQ716564
	c.1013 C > T	Exon 3	p.Pro338Leu	Missense		1	HiFi	OQ054252
	c.1049G > A	Exon 3	p.Arg350His	Missense		1	HiFi	OQ716570
	c.1058 A > G	Exon 3	p.His353Arg	Missense		1	NGS	OQ716573
	c.1063G > C	Exon 3	p.Ala355Pro	Missense		1	NGS	OQ716567
WT					KLF1*01	4	NGS 3; Sanger 1	
Total						62		

### Performance of targeted NGS and PacBio HiFi sequencing

All newly discovered *KLF1* mutations obtained from NGS and HiFi sequencing were confirmed using Sanger direct sequencing and clone sequencing, showing consistent results. The target NGS achieved an average of 9,321,773 reads (1398 Mbases) per sample, with an average coverage of 302.94 × (119.41× minimum coverage and 390.20× maximum coverage). The base coverage ranged between 98.61% and 99.70% (average 99.24%). The target base coverage ≥ 30× for all the samples was higher than 97.9%. In the detection of *KLF1* and *BCAM* genes, the amplicon size was approximately 5.6 and 13.1 kb, respectively, by using PacBio amplicon sequencing. The average number of raw reads per sample was 9004 for *KLF1* and 329 for *BCAM*.

### Determination of the In(Lu) phenotype

The NGS samples did not show any mutation in the coding region of *GATA1*, indicating the absence of X-linked XS2 phenotype. In addition, no mutation in the *BCAM* gene responsible for Lu_null_ was found in the NGS samples (Fig. 3). Therefore, all NGS samples should be categorized as the In(Lu) phenotype, although three samples did not exhibit any functional mutation in the *BCAM*, *GATA1*, and *KLF1* genes.

Similarly, in HiFi samples, no inactivating *BCAM* mutation was identified (Fig. 3). All HiFi samples carried known *KLF1* mutations in the In(Lu) phenotype or new mutations resulting in haploinsufficiency or heterozygous mutations leading to amino acid substitution of conserved residues in the zinc finger domains, which are two of the main mechanisms underlying In(Lu) (Table 1).

Given the poor DNA quality, only sequencing of the *KLF1* gene was performed by the Sanger method on seven samples. Amongst them, three samples harboured a frameshift mutation that was already known, whereas two had new mutations that were in line with the ones identified by NGS. Additionally, one sample displayed a new mutation at the base position 895 (c.895 C > G), and c.895 C > T was previously identified as a mutation associated with the In(Lu) phenotype. Therefore, the above six samples could be confirmed as having the In(Lu) phenotype. However, the one remaining sample did not have *KLF1* mutations that are known to cause the In(Lu) phenotype, whereas Lu_null_ or X-linked XS2 phenotype could not be excluded.

In summary, of the 62 samples that were tested, 61 were identified as having the In(Lu) phenotype. However, the lack of LU antigens in one sample remains unclear.

### Haplotypes of novel mutations

In particular, the *KLF1* haplotypes were determined in all the samples carrying new mutations. For cases where new mutations were identified through NGS or Sanger sequencing but were found to carry other heterozygous polymorphism sites in different *KLF1* exons, determining the haplotype was a challenge. Here, this challenge was resolved by utilizing HiFi detection to determine the haplotypes. The detailed information of the haplotypes is listed in Table 2.

**Table 2:** *KLF1* haplotypes in samples carrying novel *KLF1* mutations

Sample No.	KLF1 mutation	Haplotype 1	Haplotype 2*	Predicted by
1	c.3G > A	c.3G > A; c.304T > C	KLF1*BGM12	Clone sequencing
2	c.70_71delCA	c.70_71delCA	KLF1*01	Clone sequencing
3	c.87 + 12_87 + 25delAAGGTGGGGTCTAG	c.87 + 12_87 + 25delAAGGTGGGGTCTAG; c.304T > C	KLF1*BGM12	Clone sequencing
4	c.207_217delGGACGCCACCT	c.207_217delGGACGCCACCT; c.304T > C	c.325 C > T	Clone sequencing
5	c.406delG	c.304T > C; c.406delG	KLF1*BGM12	Clone sequencing
6	c.406delG	c.304T > C; c.406delG	KLF1*BGM12	Clone sequencing
7	c.417_421delCCTGC	c.417_421delCCTGC	KLF1*01	Clone sequencing
8	c.839delC	c.304T > C; c.839delC	KLF1*BGM12	Clone sequencing
9	c.880_881insACACCAAGAGCT	c.304T > C; c.880_881insACACCAAGAGCT	KLF1*BGM12	Clone sequencing
10	c.880T > G	c.304T > C; c.880T > G	KLF1*01	Clone sequencing
11	c.895 C > G	c.304T > C; c.895 C > G	KLF1*01	Clone sequencing
12	c.916G > A	c.304T > C; c.916G > A	KLF1*BGM12	Clone sequencing
13	c.916G > A	c.916G > A	KLF1*01	Clone sequencing
14	c.953G > A	c.953G > A	KLF1*BGM12	HiFi sequencing
15	c.982delC	c.304T > C; c.980delC	KLF1*01	HiFi sequencing
16	c.982delC	c.304T > C; c.980delC	KLF1*BGM12	Clone sequencing
17	c.982 C > T	c.982 C > T	KLF1*01	Clone sequencing
18	c.1010G > A	c.1010G > A	c.304T > C; c.544T > C	HiFi sequencing
19	c.1010G > T; 1011 C > T	c.304T > C; c.1010G > T; 1011 C > T	KLF1*BGM12	Clone sequencing
20	c.1012 C > A	c.325 C > T; c.1012 C > A	KLF1*BGM12	HiFi sequencing
21	c.1012 C > A	c.325 C > T; c.1012 C > A	KLF1*01	HiFi sequencing
22	c.1013 C > T	c.1013 C > T	KLF1*BGM12	HiFi sequencing
23	c.1049G > A	c.304T > C; c.1049G > A	KLF1*BGM12	Clone sequencing
24	c.1058 A > G	c.304T > C; c.1058 A > G	KLF1*01	HiFi sequencing
25	c.1063G > C	c.304T > C; c.1063G > C	KLF1*01	HiFi sequencing

* KLF1*BGM12 is c.304T > C (normal phenotype); KLF1*01 is common allele (wild type) [9]

### Characteristics of the new missense mutations

**Fig. 4:**
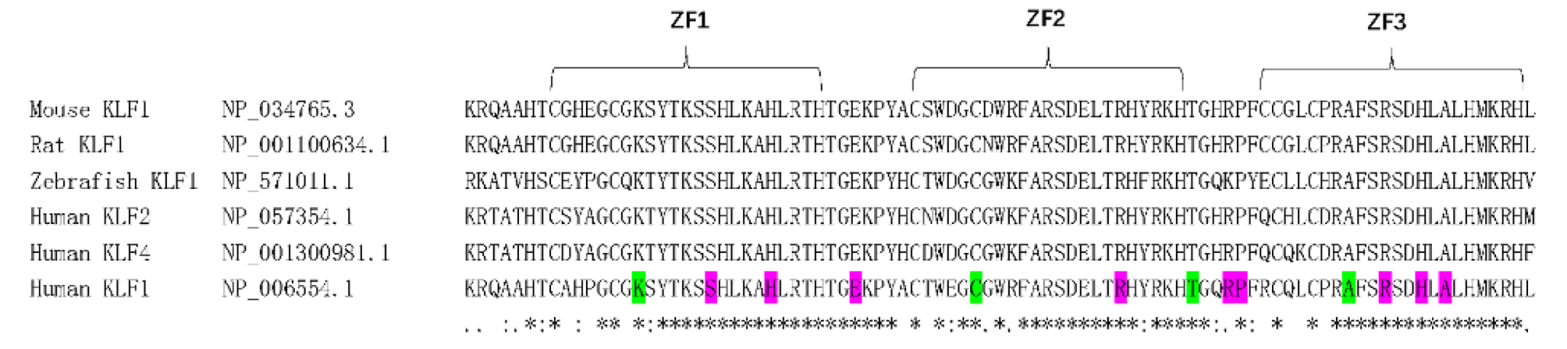
Sequence alignment of the positions of missense mutations in the zinc finger domains between KLF1 in different species and KLF family members. Green, known mutations; purple, new mutations

Mouse, rat, zebrafish and human KLF1 protein sequences, as well as human KLF2 and KLF4, which have a relatively close evolutionary relationship with KLF1, were aligned with CLUSTAL multiple-sequence alignment program [11]. By sequence alignment, the majority of missense mutations identified in this work were situated in the conserved regions of the KLF1 zinc finger structure or zinc finger linkers, with only a few exceptions (Fig. 4).

## Discussion

KLF1 is a master regulator of erythropoiesis, which plays an essential role in the activation of adult β-globin expression and regulates around 700 downstream genes that are responsible for various biological functions [8]. Since the first naturally occurring mutation reported in individuals with In(Lu), numerous KLF1 mutations have been discovered. However, mutations in KLF1, which can be classified into four distinct groups, can result in a variety of red cell phenotypes and a wide range of red blood cell disorders [8]. Class 1 mutations have little to no impact on function, whereas class 2 and 3 mutations can result in weakened or lost function [8]. In particular, class 4 mutation is a specific dominant variant (p.E325K) [8]. In recent years, some studies have found that homozygous or compound heterozygous class 2/3 mutation in KLF1 might contribute to severe haemolytic anaemia [12–15]. Considering that the pathogenesis of a haemolytic disease is complex, which involves multiple genes in most cases, identifying clinically relevant class 2 and class 3 mutations of KLF1 from specific phenotypic variation with a clear genotype–phenotype correlation is important [8]. Moreover, identifying variants in KLF1 will help define its functionally active domains and understand the broad range of phenotypes these variants produce [16].

In(Lu) is a rare blood group that is highly sensitive to the level of functional KLF1. Individuals with In(Lu) have one normal *KLF1* allele and another allele with a class 2 or 3 *KLF1* variant, indicating that they only have one copy of the clinically significant hypomorphic or loss-of-function KLF1 mutation [6]. Consequently, the In(Lu) phenotype serves as a useful marker for identifying KLF1 mutations and as a powerful model for studying related mechanisms. For example, a study reported in 2021 revealed that four patients diagnosed with severe neonatal haemolytic anaemia carried compound heterozygous mutations of *KLF1* gene [13]. All patients had c.519_525dupCGGCGCC on one allele and one class 2 mutation (c.892G > C, c.902G > A or c.1003G > A) on the other allele. Amongst them, three alleles, KLF1*BGM34 (c.519_525dupCGGCGCC), KLF1*BGM58 (c.892G > C) and KLF1*BGM70 (c.1003G > A), have been confirmed to cause the In(Lu) phenotype [9]. Notably, two novel mutations (c.895 C > G and c.1012 C > A) identified in this study have been previously reported in patients with β-thalassemia and borderline haemoglobin A2 that might increase HbF production, thereby improving the clinical severity of β-thalassemia [17, 18]. However, at present, no literature on the phenotype and biological function of these two mutations when they occur independently has been found. Our results support the hypothesis that these two mutations may be class 2 mutations of KLF1.

Apart from being a useful marker, in clinical practice, blood products from donors with the In(Lu) phenotype would be appropriate for transfusion to patients with matching phenotypes [6]. This approach can reduce the risk of alloimmunisation and transfusion reactions, considering that In(Lu) is the most common type of serological Lu(a − b−) phenotype [10]. However, the In(Lu) phenotype is observed at a very low frequency. The study of the genetic mechanism of the In(Lu) phenotype primarily relies on case reports, with only a limited number of population-based studies available. To date, there have been only three population-based studies that have included more than 20 In(Lu) samples. Specifically, these studies involved 24, 79, and 120 In(Lu) individuals, respectively [7, 19, 20]. Based on the current data, it has been indicated that the In(Lu) phenotype occurs at approximately 0.02% in Japanese populations and between 0.005% and 0.032% in England and Wales [10, 20]. In a previous study conducted on a relatively small population, the frequency of In(Lu) was approximately 0.02% in the Chinese population [21]. Therefore, pre-screening blood donors with In(Lu) and storing their blood as a rare blood resource to benefit blood transfusions are important. Understanding the molecular mechanism of In(Lu) can promote the screening and identification of In(Lu) blood donors through cost-effective genetic testing, in the context of the low incidence rate of In(Lu) and high cost of serotyping. To date, 71 types of KLF1 mutation resulting in the In(Lu) phenotype were summarized and published on the official website of the International Society of Blood Transfusion (ISBT) [9]. This study has identified up to 21 new and eight previously known mutations in KLF1, which significantly enhances our knowledge of KLF1 variants that contribute to In(Lu).

Our data reveal the following genetic characteristics of KLF1 in Chinese In(Lu) individuals. Firstly, consistent with our previous reports, the main mutation site is c.519_525dupCGGCGCC (p.Gly176Argfs*179), which represents approximately 37% of the total number of samples tested [21]. Given the significant number of KLF1 mutations and their distribution, this mutation must be considered as the primary genetic screening strategy in China. Secondly, class 2 mutations detected in this study are primarily located in the conserved KLF1 zinc finger domain, with few exceptions, indicating the importance of the conserved functional domain of zinc finger structures. Several mutations are located within the linker region of the zinc finger structure, which is a relatively rare occurrence in previous reports [9]. This finding suggests that the zinc finger domain and the linker region are biologically active. Thirdly, similar to previous studies, our study has confirmed that three individuals with In(Lu) exhibit no functional KLF1 mutations, indicating the presence of additional regulatory mechanisms [7, 20].

The application of targeted NGS and long-read HiFi sequencing in investigating the genetic background of In (Lu) individuals has not been reported before. In this study, we compared these two high-throughput technologies with the Sanger sequencing method as follows. All three sequencing technologies used in this study demonstrate high accuracy. Sanger sequencing remains the gold standard for determining DNA sequences, with low cost for single detection. However, when multiple genes and targets need to be detected simultaneously, the cost of Sanger sequencing increases significantly. The NGS panel utilized in this study provides a considerably wider range of detection compared to Sanger sequencing. Additionally, the cost of detecting a single target gene is lower with NGS panel than with Sanger sequencing. Other benefits of the NGS approach in this study include its design to incorporate known 50 blood group genes and two primary erythroid transcription factors, namely KLF1 and GATA1. This design allows for the exclusion of Lu_null_ and X-linked XS2 phenotypes, which are indistinguishable from the In(Lu) phenotype when using serologic methods. On the other hand, the In(Lu) phenotype is characterized by a significant decrease in the expression of LU antigens, as well as varying degrees of reduced expression of other blood group antigens. Unlike LU antigens, the other specific antigens affected and the extent of their decrease can vary significantly among individuals. By comparing the serologic and NGS results, it is possible to determine whether these reductions are due to abnormalities in the encoding genes or abnormalities in the regulation during erythroid differentiation caused by KLF1 mutations. However, distinguishing haplotypes in target regions and detecting complex structural variations can be challenging when using Sanger and NGS technologies. In this study, we effectively analysed the *KLF1* haplotypes by utilizing PacBio HiFi sequencing technology, particularly when the cloning sequencing method was unable to provide a solution. Clarifying KLF1 haplotypes can help identify In(Lu)-associated *KLF1* alleles and provide novel evidence of benign KLF1 variants, such as c.544T > C found in sample 18 [6, 9, 22]. Moreover, as KLF1 acts as a master regulator of erythropoiesis, the severity of the clinical phenotype is influenced by the specific type and class of the KLF1 mutations, as well as whether the mutations are monoallelic or biallelic [8]. In cases where KLF1 mutations are suspected to be associated with pathological conditions, it is crucial to accurately determine the *KLF1* haplotype, especially when multiple class 2 or class 3 mutations are present. This is because compound heterozygous KLF1 mutations that impact function often result in more severe clinical complications in most cases [8, 12–15]. However, it should be noted that Hifi sequencing currently has a higher cost compared to NGS. Therefore, this study focused solely on the key *KLF1* and *BCAM* genes when using Hifi sequencing, providing limited blood type gene information. Overall, our work demonstrates the advantages of high-throughput sequencing technologies over Sanger sequencing in detection of genetic background of In(Lu). Targeted NGS offers a wider detection range and lower comprehensive cost. PacBio Hifi sequencing provides accurate haplotype confirmation. As the cost of Hifi sequencing technology decreases, its superior capabilities in detecting structural variations and confirming haplotypes will lead to its wider application.

This research also has a few limitations that should be addressed in future studies. One limitation of this study is the delay between DNA collection and analysis, which prevented the detection of other blood group antigens on red blood cells apart from LU. Thus, further research should be conducted to determine the impact of newly discovered mutations, particularly those in class 2, on other blood group antigens. In addition, this study has identified two atypical *KLF1* mutants (start loss and splice site mutations). There are few reported cases of splice site mutations associated with In(Lu) while no start loss mutation has been reported [9, 23]. Further verification of more samples and additional functional studies are necessary to confirm their roles definitively. For example, start codon loss is commonly considered a loss-of-function mutation, resulting in the silencing of a particular gene [24, 25]. Additionally, there have been a few instances where this type of mutation has been linked to reduced protein expression in blood group antigens [26]. Therefore, the start codon variant (c.3G > A) identified in this study might be classified as a class 2 or class 3 mutation. Moreover, the mechanism of reduced expression of LU antigens in four individuals was not clarified. One plausible explanation is that other genes might be involved in the regulation of LU expression. For instance, previous studies have found that monoallelic defective mutations in SUPT5H may result in reduced expression of KLF1 during erythrocyte differentiation, suggesting its potential role in LU regulation [27]. Including other candidate genes, such as *SUPT5H*, in the genetic screening of individuals with the In(Lu) phenotype might provide further valuable information.

## Conclusions

In conclusion, we primarily utilised targeted NGS and HiFi sequencing to verify the In(Lu) phenotype and identify a diverse array of known and previously undiscovered KLF1 mutations in In(Lu) individuals. The two high-throughput sequencing methods expanded the scope of detection of the blood group genes and demonstrated high accuracy. Also, the distribution of KLF1 variants in Chinese In(Lu) individuals is revealed for the first time from population-based data, which might contribute to clinical transfusion. The discovery of 21 new KLF1 mutations offers a potential explanation for erythrocyte disorders and hemoglobin abnormalities that have previously been unexplained, such as rare hemolytic anemias. By conducting further research on their clinical significance and biological function, these mutations could potentially be utilized as targets for detecting related diseases.

## Methods and materials

### Subjects

The screening of Lu(a-b-) individuals was conducted using anticoagulated whole blood samples collected from voluntary unpaid blood donors in the Shanghai Blood Center over the course of the past 10 years. Informed consent was obtained from all donors. The blood samples were randomly obtained from unrelated eligible donors aged between 18 and 55, who passed the routine tests for blood types and transfusion-transmitted infections. Anti-Lu^b^ (BRIC108) was obtained from the International Blood Group Reference Lab (IBGRL, Bristol, UK) and used to screen for Lu^b^-negative blood samples by conventional serotyping methods. In addition, anti-Lu^a^ (213553, Grifols, Spain) was utilised to eliminate Lu(a + b−) samples from the screened Lu^b^-negative samples, following the manufacturer’s instructions. Serological Lu(a − b−) samples were selected as research subject. Genomic DNA was manually extracted from all serological Lu(a − b−) samples using the QIAamp DNA Blood Mini Kit (Qiagen, Germany), following the provided instructions. The extracted DNA samples were then kept frozen for further analysis.

### Sequencing workflow

DNA quality was assessed to determine the follow-up appropriate gene sequencing method. In brief, DNA concentration was measured using a NanoDrop 2000 C spectrophotometer (NanoDrop Technologies, USA), and DNA integrity was verified through gel electrophoresis. Samples with good DNA integrity and high concentration were preferentially detected by targeted NGS. The requirements for NGS library construction included a total amount of DNA greater than 500 ng, an OD260/280 ratio between 1.6 and 2.0 and a main band obtained from DNA electrophoresis greater than 10 kb. Samples that do not meet the requirements of targeted NGS sequencing but meet the requirements of HiFi sequencing were analysed by HiFi sequencing. For PacBio HiFi sequencing, the DNA concentration was greater than 10 ng/µL, and the main band of DNA was intact. Poor-quality samples in which high-throughput sequencing could not be performed were subjected to Sanger sequencing. All sequencing methods are described in detail below.

### Targeted NGS

The customized NGS panel was designed and built to cover the whole gene region plus 10 kb upstream and downstream for all known 50 blood group genes, as well as coding genes of two primary erythroid transcription factors, namely, *KLF1* and *GATA1* (Twist Bioscience, USA) [9]. For NGS library construction, only DNA samples that met the quality control requirements were used. NGS library construction was completed using the DNA Library Prep Kit (Twist Bioscience, USA). The customized panel consisting of 52 genes was used for hybrid capture. Paired-end sequencing was conducted on the DNBSEQ-T7 sequencing platform (MGI, China) with PE150 mode by Shanghai WeHealth BioMedical Technology Co. (Shanghai, China). Quality control of raw data was performed using FASTP, followed by the removal of low-quality reads. The threshold for removal was set at a Q30 score of 85%. The aligned sequences were mapped to the human reference genome (GRCh38/hg38) using BWA. Subsequently, SNP and Indel variations were analysed using SAMtools and GATK after deduplication and base calibration. The detected variants were annotated using Annovar, which included population frequency databases such as gnomAD, OMIM and HGMD.

### PacBio HiFi sequencing

Samples that did not meet NGS quality control requirements but met PacBio HiFi sequencing quality control requirements were subjected to targeted amplification of the *BCAM* and *KLF1* genes using specific primers. Sequencing was performed on the PacBio Sequel II platform by Xi’An Haorui Genomics Technologies Ltd. The entire *KLF1* gene plus 1.3 kb upstream and 1.5 kb downstream of the coding region, as well as the entire *BCAM* gene plus 600 bp upstream and 200 bp downstream, were specifically amplified by KLF1-F3/KLF1-R3 and BCAM-F3/BCAM-R1 (see Additional file 2), respectively. After data collection, SMRTlink (v10.1.0) was used to remove adapter sequences and convert the original polymerase reads into subreads. Then, CCS (v6.2.0) was used to cluster and filter subreads from the same SMRTbell sequencing template to obtain high-quality (QV > 20) consensus sequences, that is, HiFi reads. The pbaa software (v1.0.3) was used to error correct and cluster HiFi reads from different amplification regions to obtain a consensus sequence for each amplification product. The consensus sequences of the amplification products were aligned to the human reference genome CHM13v2.0 and imported into the visualization analysis tool SnapGene (v6.1.1) for analysis. In addition, for samples with known and novel polymorphisms spanning two *KLF1* exons, PacBio HiFi sequencing was performed to confirm haplotypes.

### Sanger sequencing

Samples that were found to have poor DNA quality or a low total DNA amount were excluded from high-throughput testing. These samples were instead analysed by Sanger sequencing. In brief, the promoter region, exon 1 and exon 3 of the *KLF1* gene were amplified and sequenced in accordance with the methods listed in the literature [7]. Exon 2 of *KLF1* was amplified and sequenced by using the primers KLF1ex2F and KLF1ex2R (see Additional file 2). Moreover, new mutations of the *KLF1* gene detected by targeted NGS and HiFi sequencing were further verified by Sanger sequencing. All new *KLF1* mutations were further confirmed by clone sequencing because of mixed peaks in the sequencing results. For samples carrying more than one heterozygous *KLF1* polymorphic site in a single exon, clone sequencing was performed to determine the haplotype. PCR amplification was carried out using KOD-Plus-Neo DNA polymerase (Toyobo, Japan). Primer synthesis and Sanger sequencing were performed by the Beijing Genomics Institute (China).

### Bioinformatic analysis

All newly discovered *KLF1* mutations were submitted to the NCBI database after being compared with the *KLF1* Allele table v1.0 30-JUN-2021, which is listed on ISBT’s official website [9]. The protein sequences of KLFs were obtained from GenBank. The amino acid sequences of zinc finger domains and neighbouring amino acids of KLF1 in different species, as well as human KLF2 and KLF4, were subjected to multiple sequence alignment utilising Clustal Omega program [28].

### Electronic supplementary material

Below is the link to the electronic supplementary material.

Supplementary Material 1

Supplementary Material 2

Supplementary Material 3

## Data Availability

The datasets generated and analysed during the current study are available in the NCBI repository under the accession numbers: OQ054248 to OQ054253, OQ716563 to OQ716573, and OR000212 to OR000215.
