# Supplementary material for: Targeted next-generation sequencing and long-read HiFi sequencing provide novel insights into clinically significant KLF1 variants
Source: BMC Genomics. 2024 Mar 1;25:230. doi: 10.1186/s12864-024-10148-x (PMC10908068; doi:10.1186/s12864-024-10148-x)
Supplement: Supplementary file 1 — Supplementary Material 1 [file 12864_2024_10148_MOESM1_ESM.pptx]

## Slide 1
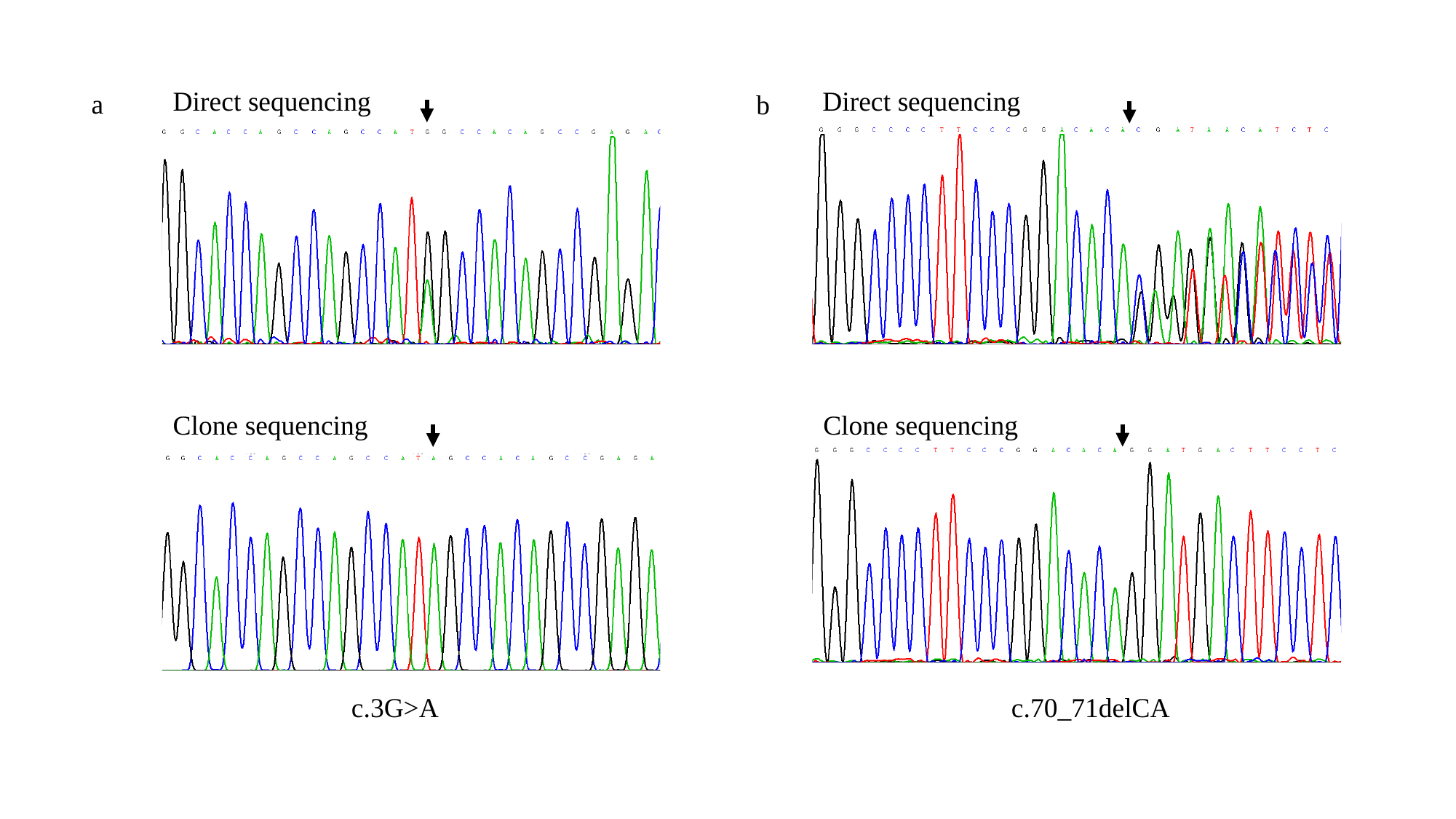

Direct sequencing
Direct sequencing
a
b
Clone sequencing
Clone sequencing
c.70_71delCA
c.3G>A

## Slide 2
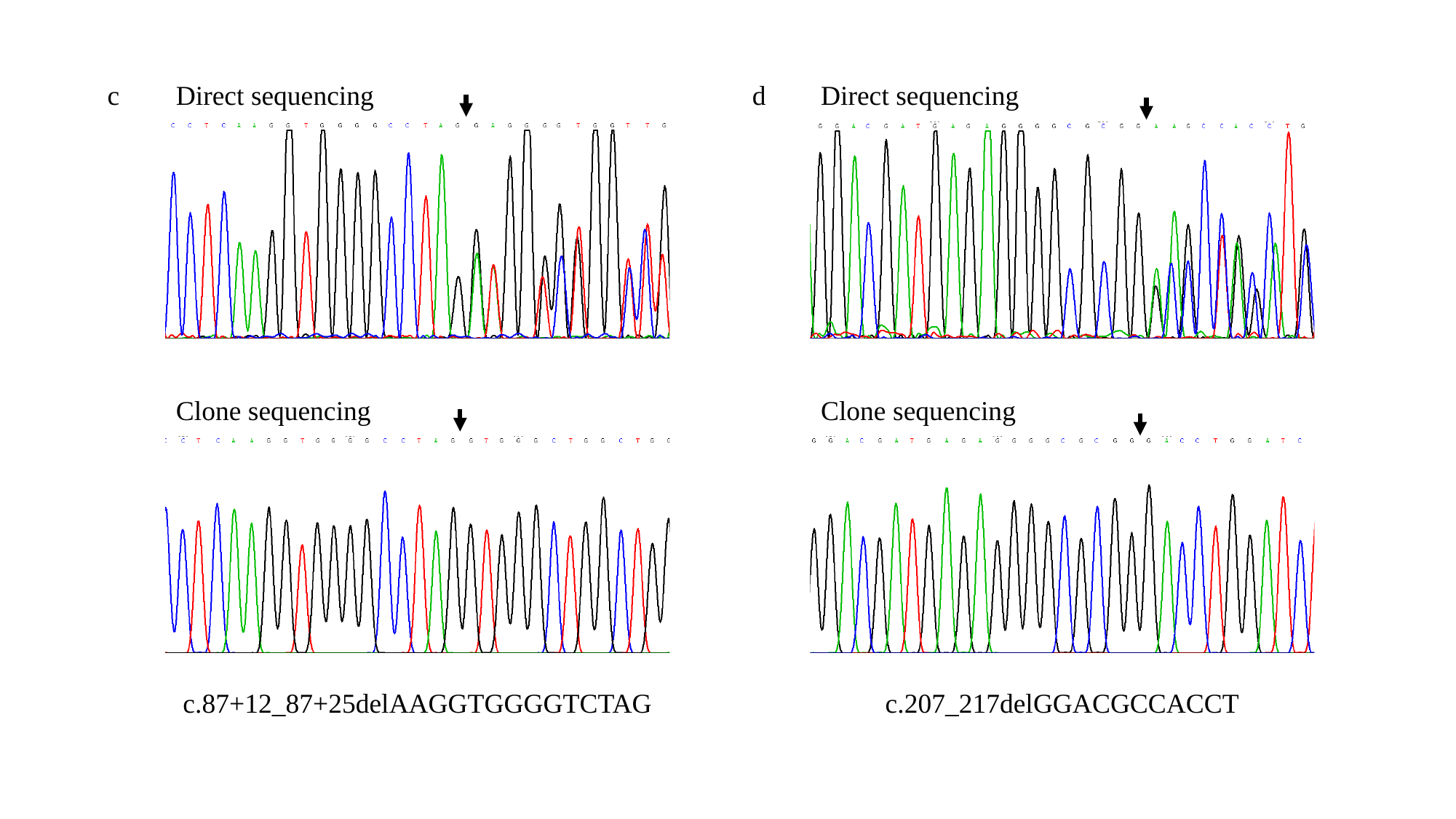

d
Direct sequencing
c
Direct sequencing
Clone sequencing
Clone sequencing
c.87+12_87+25delAAGGTGGGGTCTAG
c.207_217delGGACGCCACCT

## Slide 3
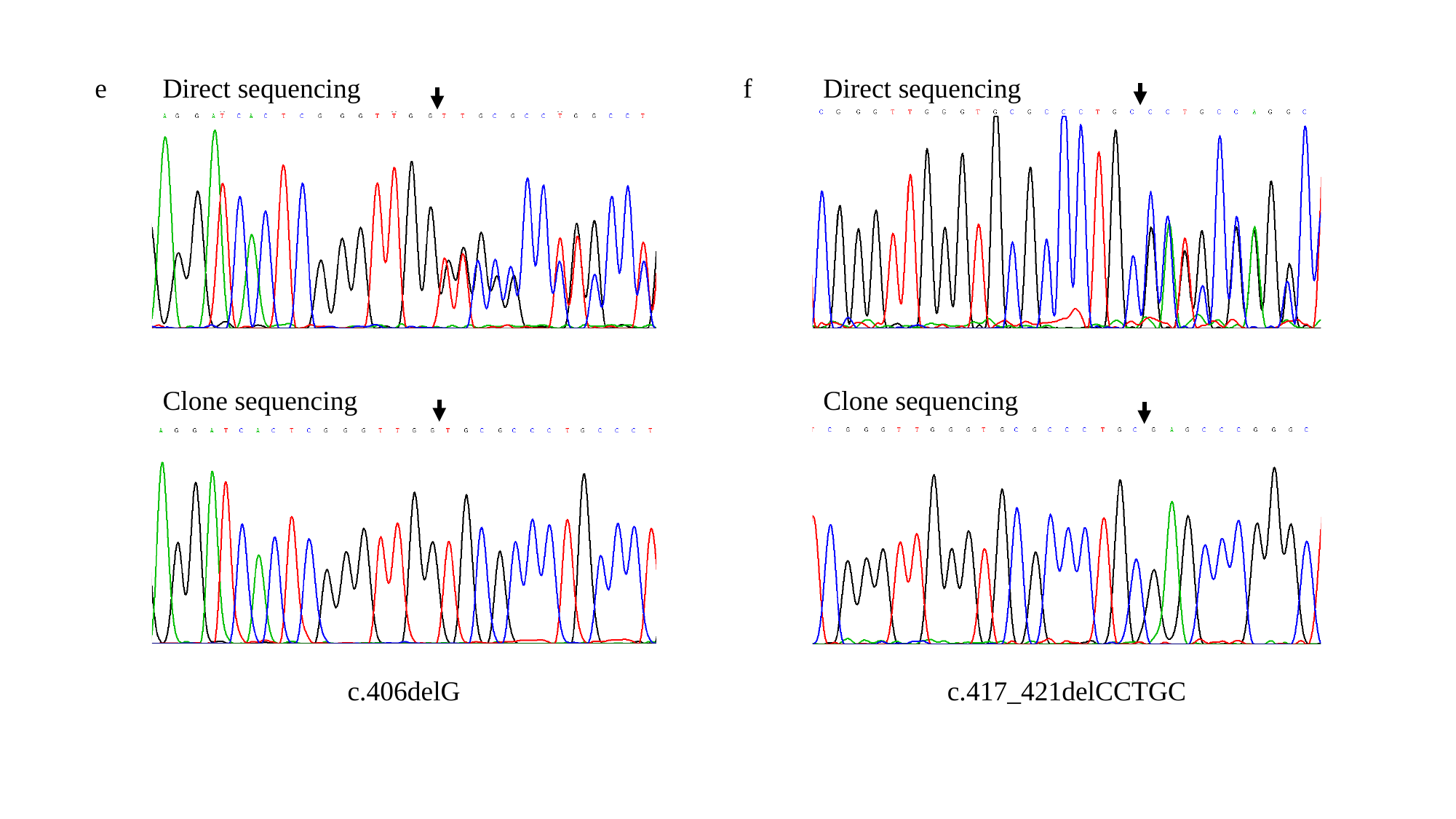

e
Direct sequencing
f
Direct sequencing
Clone sequencing
Clone sequencing
c.406delG
c.417_421delCCTGC

## Slide 4
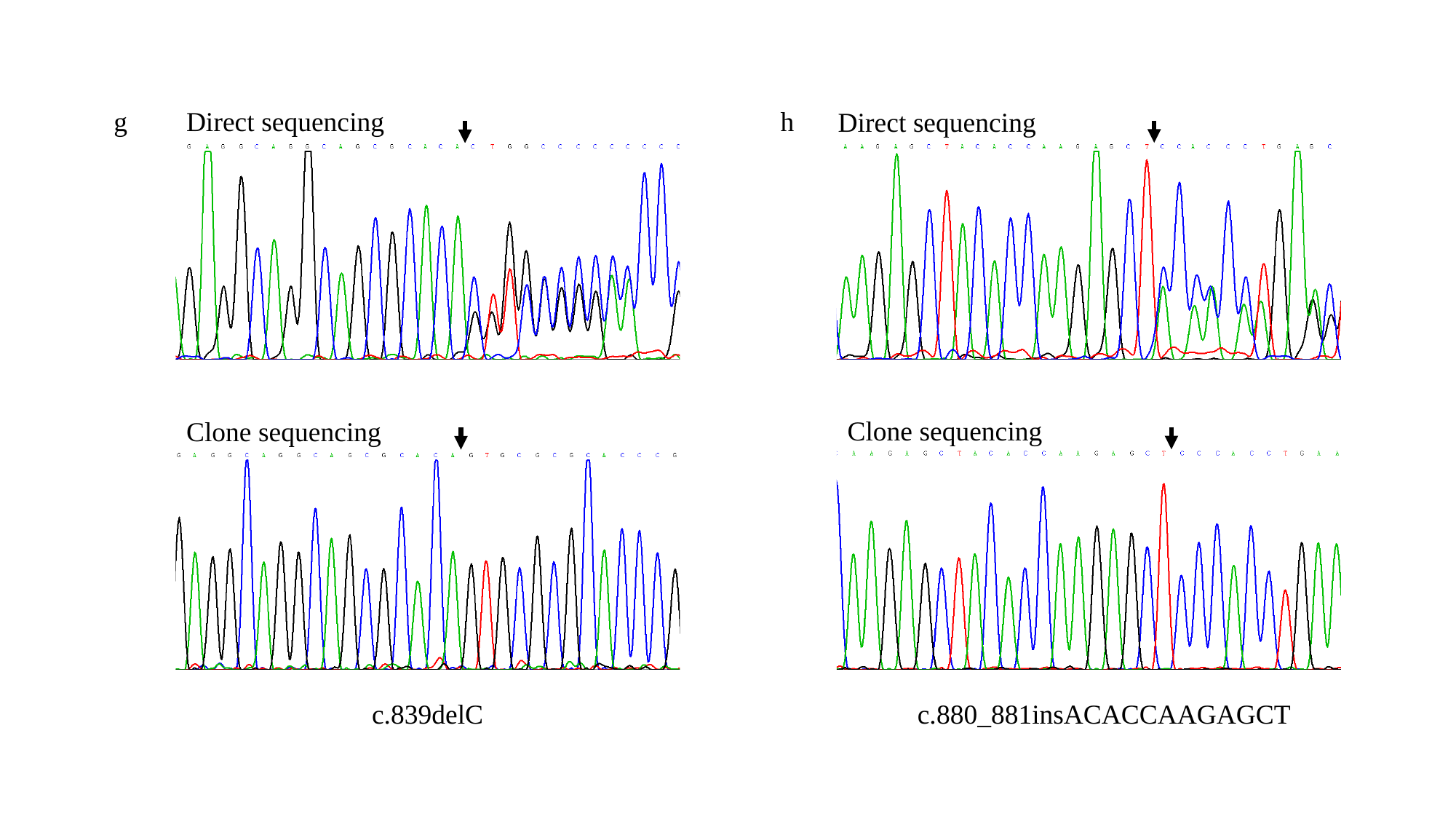

h
g
Direct sequencing
Direct sequencing
Clone sequencing
Clone sequencing
c.839delC
c.880_881insACACCAAGAGCT

## Slide 5
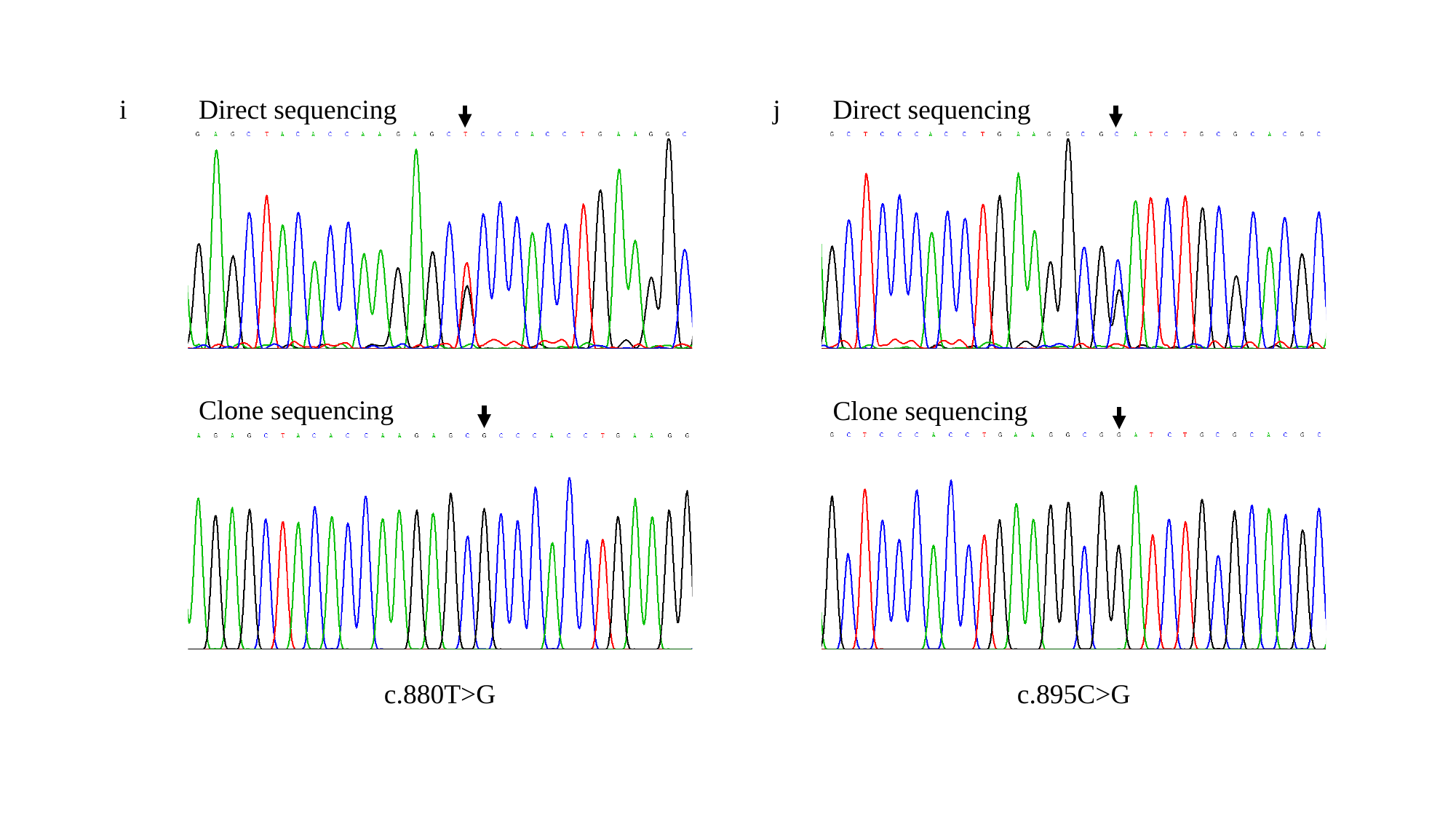

Direct sequencing
j
i
Direct sequencing
Clone sequencing
Clone sequencing
c.880T>G
c.895C>G

## Slide 6
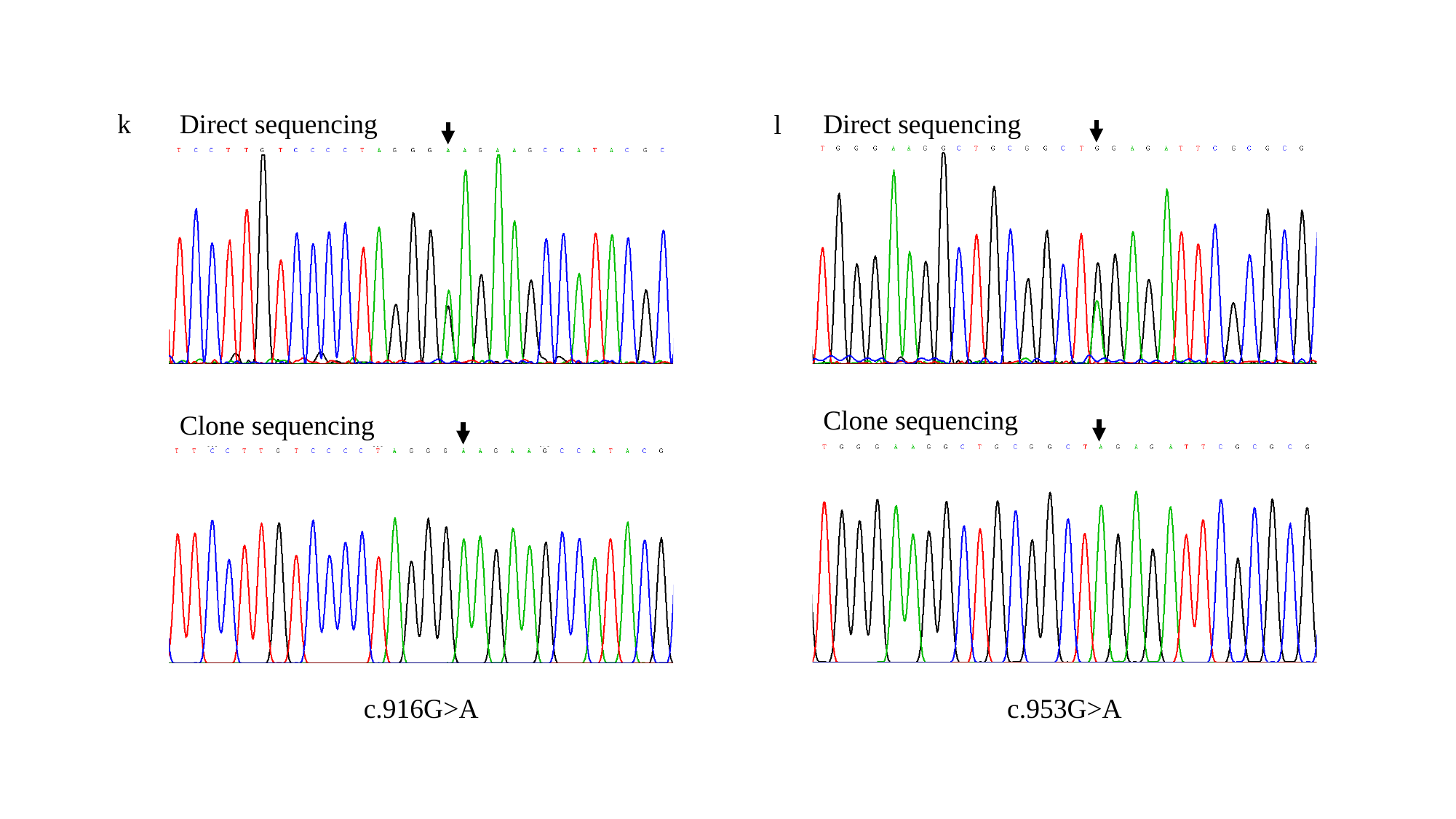

k
Direct sequencing
Direct sequencing
l
Clone sequencing
Clone sequencing
c.916G>A
c.953G>A

## Slide 7
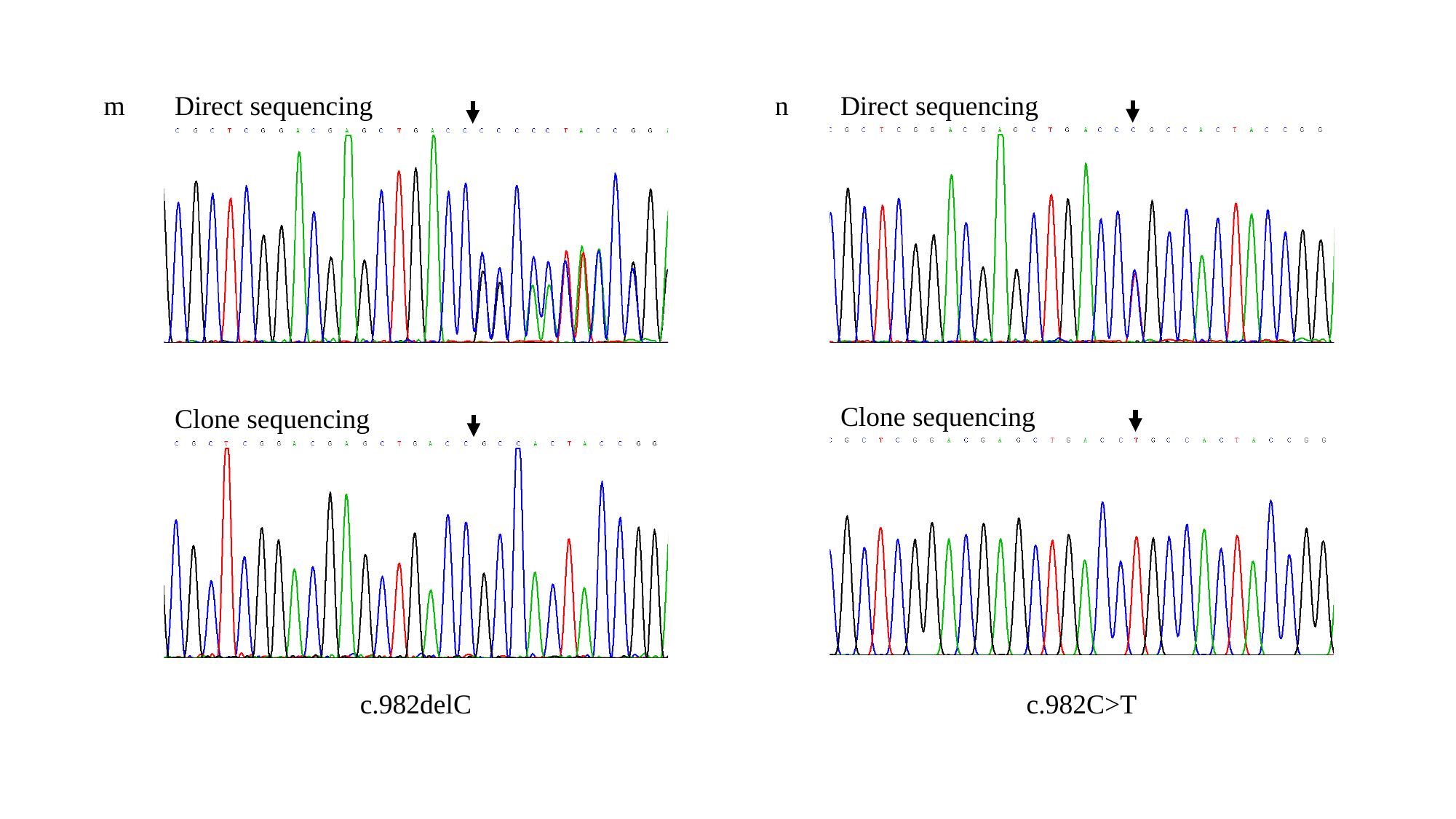

m
Direct sequencing
n
Direct sequencing
Clone sequencing
Clone sequencing
c.982C>T
c.982delC

## Slide 8
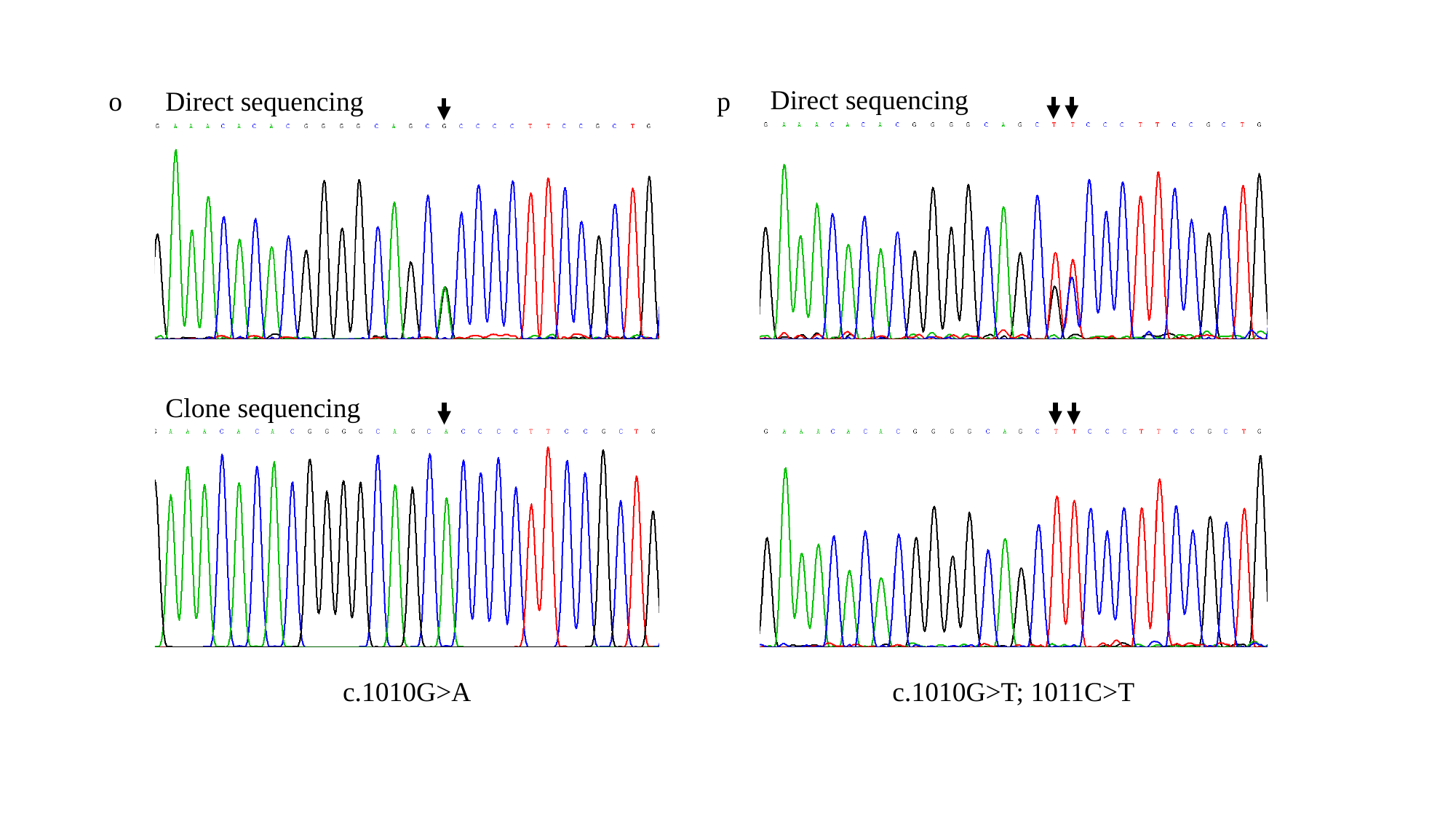

Direct sequencing
o
Direct sequencing
p
Clone sequencing
c.1010G>A
c.1010G>T; 1011C>T

## Slide 9
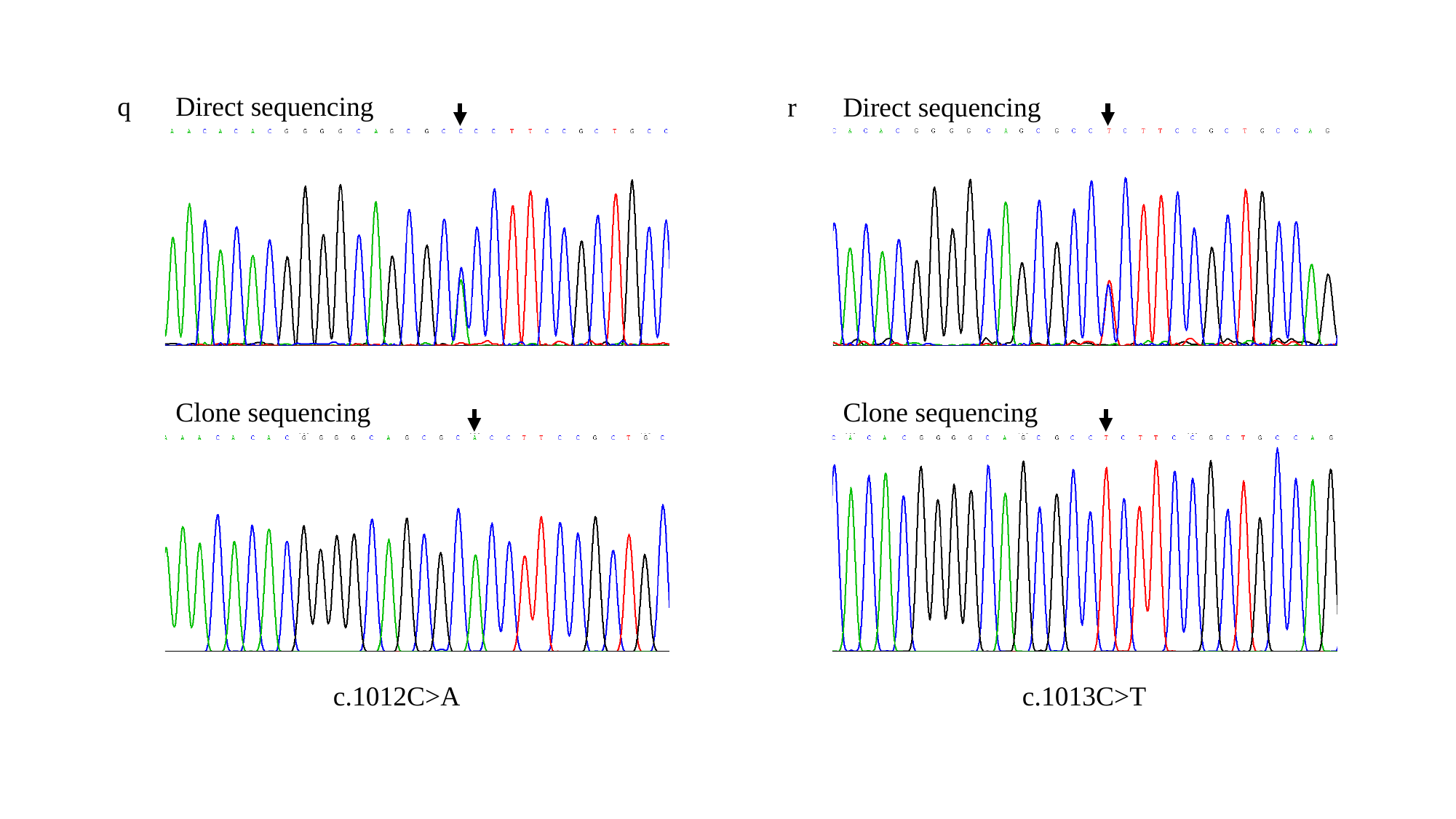

q
Direct sequencing
r
Direct sequencing
Clone sequencing
Clone sequencing
c.1012C>A
c.1013C>T

## Slide 10
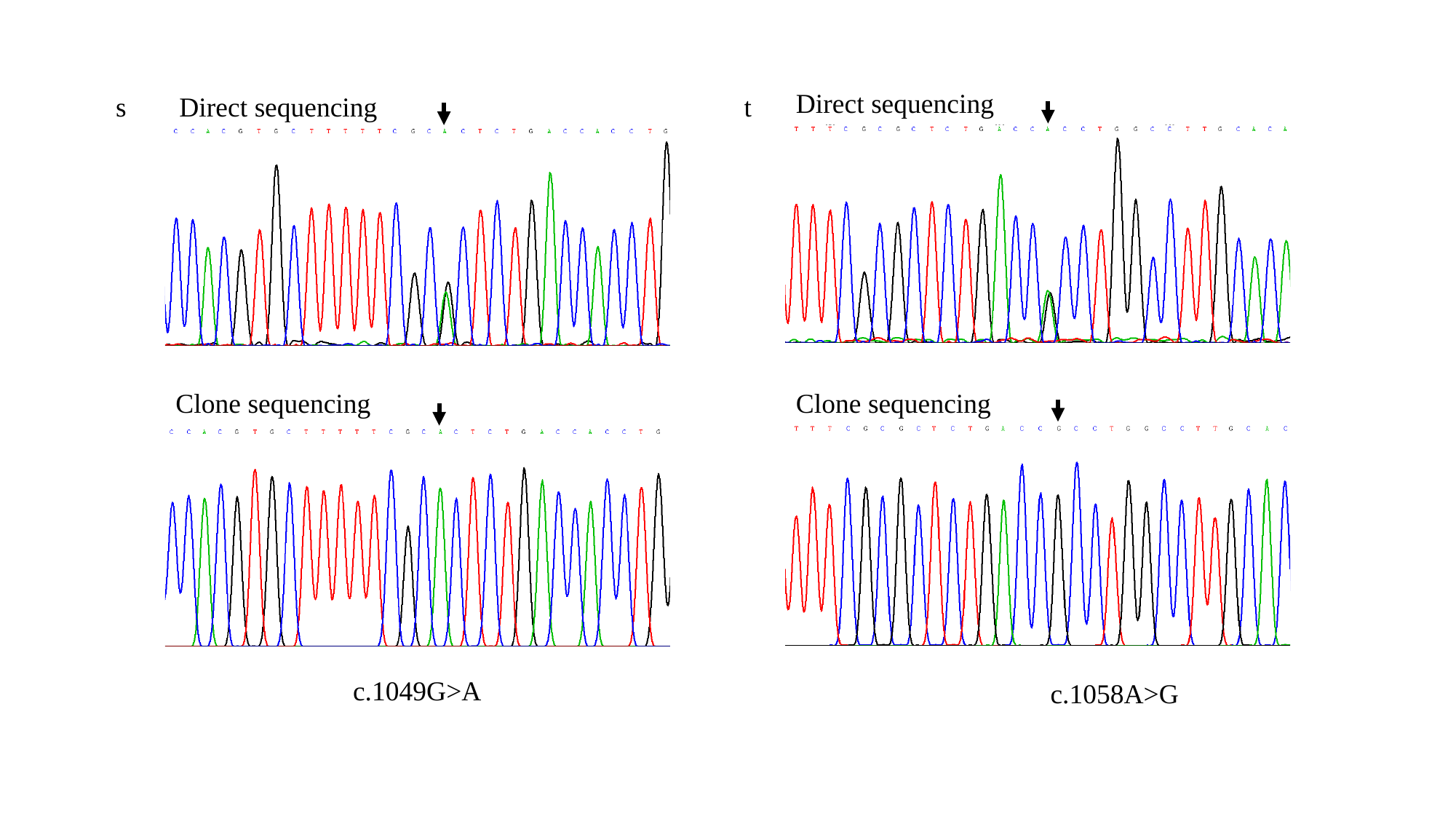

Direct sequencing
Direct sequencing
t
s
Clone sequencing
Clone sequencing
c.1049G>A
c.1058A>G

## Slide 11
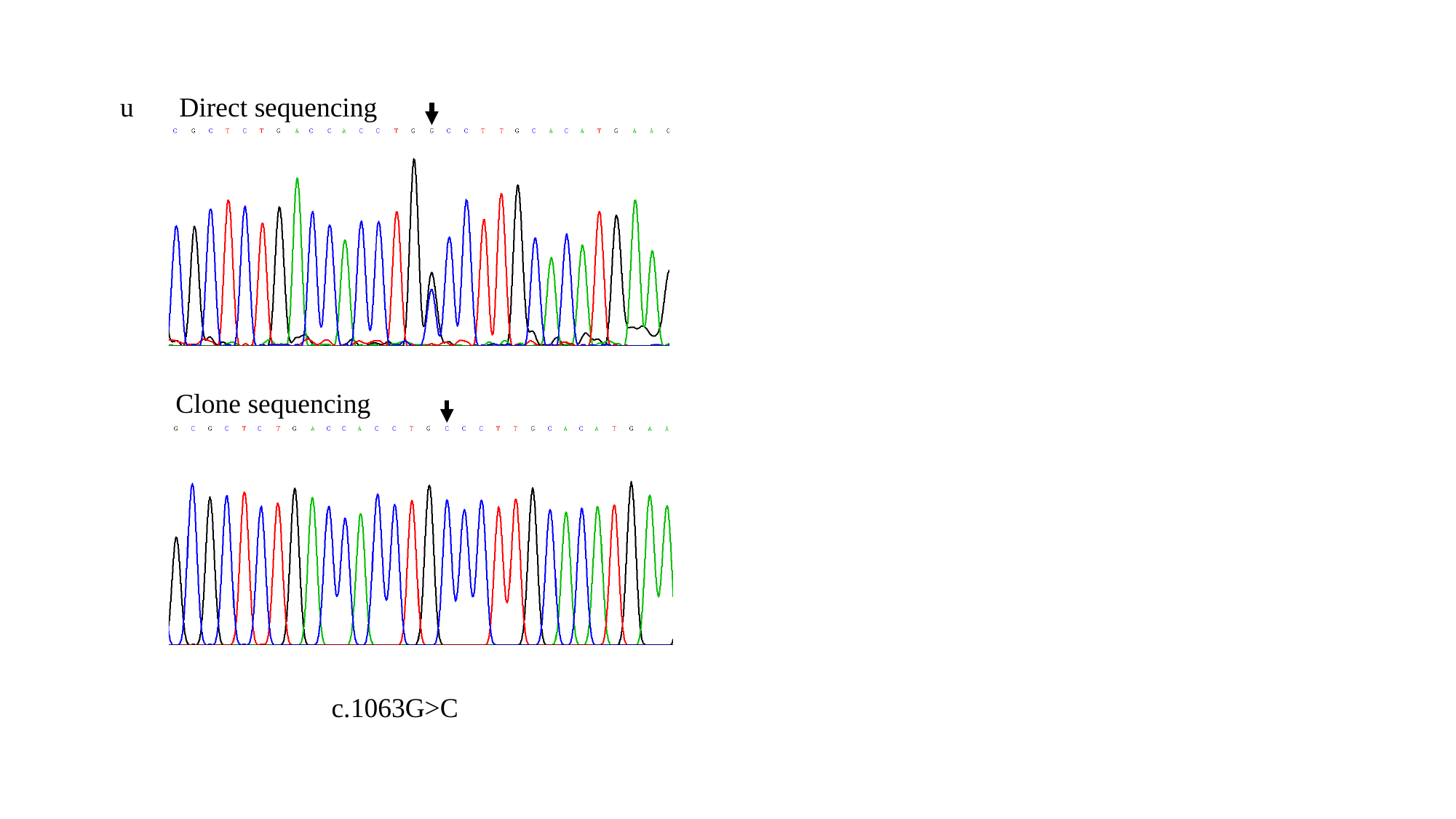

u
Direct sequencing
Clone sequencing
c.1063G>C
