# Supplementary material for: Targeted next-generation sequencing and long-read HiFi sequencing provide novel insights into clinically significant KLF1 variants
Source: BMC Genomics. 2024 Mar 1;25:230. doi: 10.1186/s12864-024-10148-x (PMC10908068; doi:10.1186/s12864-024-10148-x)
Supplement: Supplementary file 2 — Supplementary Material 2 [file 12864_2024_10148_MOESM2_ESM.docx]

Supplementary Table 1. Primers used in this study

| Primer ID | Primer Sequence (5’ to 3’) |
| --- | --- |
| KLF1ex2F | GAGCGGGGAGAGGGGCGGTTAG |
| KLF1ex2R | CGTGCAGGCGTATGGCTTCTCC |
| KLF1-F3 | CTTGCTTGGTCATTCCTGGAGTGGGG |
| KLF1-R3 | CAGAGAGAGGGCTTCATCCTGTTGGC |
| BCAM-F3 | GAGGACCTGGATTTGAAGACCATTGA |
| BCAM-R1 | TGGGATTACAAGAATGAACCACGGCG |
