# Supplementary material for: Targeted next-generation sequencing and long-read HiFi sequencing provide novel insights into clinically significant KLF1 variants
Source: BMC Genomics. 2024 Mar 1;25:230. doi: 10.1186/s12864-024-10148-x (PMC10908068; doi:10.1186/s12864-024-10148-x)
Supplement: Supplementary file 3 — Supplementary Material 3 [file 12864_2024_10148_MOESM3_ESM.docx]

**Additional Materials**

Additional file 1

File format: .pptx

Title of data: Supplementary Figure 1.

Description of data: The sequencing peak maps of 21 novel KLF1 variants. The arrows point out the location of missense mutations or initiation of the frameshift mutations.

Additional file 2

File format: .docx

Title of data: Supplementary Table 1.

Description of data: Primers used in this study.
